# Supplementary material for: Bird wings act as a suspension system that rejects gusts
Source: Proc Biol Sci. 2020 Oct 21;287(1937):20201748. doi: 10.1098/rspb.2020.1748 (PMC7661293; doi:10.1098/rspb.2020.1748)
Supplement: Supplemental information [file rspb20201748supp1.docx]

**Supplemental information for**

**Bird wings act as a suspension system that rejects gusts.**

Jorn A. Cheney, Jonathan P. J. Stevenson, Nicholas E. Durston, Jialei Song, James R. Usherwood, Richard J. Bomphrey*, Shane P. Windsor*.

* Richard J. Bomphrey and Shane P. Windsor.

Email: rbomphrey@rvc.ac.uk and shane.windsor@bristol.ac.uk

**This portion of the PDF file includes:**

Supplementary text

Figures S1 to S6

Captions for Movies S1 to S5

Captions for Dataset S1

**Other supplementary materials for this manuscript include the following:**

Movies S1 to S5

Dataset S1

Supplementary Information Text

***Gust rejection***

We quantified the gust perturbation by its delivered impulse or, after mass-normalisation, the added vertical velocity. The effect of the perturbation could also be described by other metrics, such as a change in position or instantaneous acceleration. Impulse is clearer than position as it is not affected by variation in initial conditions, such as flight velocity. We did not select instantaneous acceleration because it neglected the previous effects of the perturbation. Expanding on this, the gust causes the bird’s torso to rise with the flow over time, as the torso rises the relative flow experienced decreases, as does the perturbation; therefore, minimising instantaneous perturbation can be achieved by performing poorly in previous time-steps, *i.e.*, early in the gust. Impulse accounts for this.

We compute impulse as

$$\int_{0}^{t} F dt$$

We separate the impulse acting on the torso into two components: the external impulse acting on the centre of mass of the system, *i.e.*, the bird; and the internal impulse acting on the torso applied within the system. The internal impulse has no effect on the centre of mass, and is a result of the bird changing shape, *i.e.*, movement of inertial appendages. The external impulse produces movement of the centre of mass, and is a result of the net aerodynamic and gravitational forces acting on the bird.

An important aspect of this approach is that it is agnostic to any specific mechanism. There are no assumptions about why or how the bird changes shape, nor does it provide any specific insight into that question. It merely separates the resultant impulses/forces.

We compute aerodynamic rejection from the difference between the expected and observed aerodynamic force. The expected force was computed from the glide simulation utilizing the aerodynamic polar derived from CFD. The observed force was computed from movement of the bird’s centre of mass.

$$\int_{0}^{t} \left( F_{CFD}-F_{CoM} \right) dt$$

$$M*\left( v_{CFD}-v_{CoM} \right)\left. \right|_{0}^{t}$$

where M is the total mass. To determine the fraction of the external impulse attributed to the torso, we normalize by the mass fraction $\frac{m_{torso}}{M}$.

$$m_{torso}*\left( v_{CFD}-v_{CoM} \right)\left. \right|_{0}^{t}$$

We compute inertial rejection as the impulse acting within the system on the torso. We’ll first discuss the logic to the approach and then discuss the physics.

The inertial rejection does not explicitly consider any effects of aerodynamic force. Inertial rejection is the difference between the expected force on the torso if the bird did not elevate its wings, and the observed force acting on the torso. If the wings did not elevate, the torso would accelerate at the same rate as the observed centre of mass:

$$F_{torso, expected}= m_{torso} a_{CoM}$$

And the observed force acting on the torso would be:

$$F_{torso, observed}= m_{torso} a_{torso}$$

The rejected inertial impulse would then be

$m_{torso}\int_{0}^{t} \left( a_{CoM}-a_{torso} \right) dt$

$$m_{torso}*\left( v_{CoM}-v_{torso} \right)\left. \right|_{0}^{t}$$

We can find that the quantity above is due to internal forces responding to movement of the wings through manipulating and differentiating the equation for the centre of mass.

$$m_{torso} x_{torso}+ m_{wing} x_{wing}= M x_{CoM}$$

$$m_{torso} a_{torso}+ m_{wing} a_{wing}= M a_{CoM}=F_{external}$$

$$m_{torso} a_{torso}+ m_{wing} a_{wing}= \left( m_{torso}+m_{wing} \right)a_{CoM}$$

$$m_{torso} a_{torso}+ m_{wing} a_{wing}= m_{torso}a_{CoM}+ m_{torso}a_{CoM}$$

$$m_{torso} \left( a_{torso}-a_{CoM} \right)+ m_{wing} \left( a_{wing}-a_{CoM} \right)= 0$$

Yielding that the internal force acting on the torso through acceleration of the wings (and Newton’s third law makes the opposite true as well) is:

$$m_{torso} \left( a_{torso}-a_{CoM} \right)= -1*m_{wing} \left( a_{wing}-a_{CoM} \right)$$

Which, as discussed above, is the inertial rejection once integrated over time.

***Glide simulation***

The equations used in the simulation are classic equations of motion. Forces were determined using aerodynamic formulas; the initial angle of attack was identified based on calculating the required pre-gust lift and solving for the angle of attack based on the CFD lift polar (Fig S3). We did not use the drag polar as initial drag values were low and were highly sensitive to error in the force estimations, *i.e.*, computing acceleration from position. The following equations and parameters describe our approach to the simulations:

| **Term** | **Description** |
| --- | --- |
| $\rho$ | Air density |
| $S$  $m$  $g$ | Planform area of geometry  Body mass  Gravitational acceleration |
| $x$  $z$ | Horizontal position along the corridor  Vertical position within corridor |
| $v$ | Velocity in global orientation |
| $\left\vert v \right\vert$  $a$ | Magnitude of flow speed  Acceleration |
| $\alpha_{0}$ | Pre-gust orientation of bird relative to horizon |
| $\Delta\alpha$ | Orientation of flow |
| $\alpha$ | Angle of attack |
| $R\left( \Delta\alpha\right)$ | Rotation matrix from flow- to global- orientation |
| $C_{l/d}$ | Coefficient of lift/drag: perpendicular and parallel to flow respectively |

Compute orientation of bird, $\alpha_{0}$, for angle of attack estimations of rigid, non-pitching model, using flight speed and vertical acceleration:

$$\left| \boldsymbol{v} \right|= \left| {\vec{\boldsymbol{v}}}_{bird}+{\vec{\boldsymbol{v}}}_{gust}\left( x \right) \right|$$

$${\vec{\boldsymbol{v}}}_{gust}\left( 0 \right)=0$$

$$solve for \alpha\boldsymbol{:}L=\frac{1}{2}\rho S\left| v \right|^{2}C_{l}\left( \alpha\right)$$

$$\Delta\alpha=\tan^{-1}(\frac{v_{z}}{v_{x}})$$

$$\alpha_{0}=\alpha-\Delta\alpha$$

As the simulation iterates, update: $position, \left| v \right|, \Delta\alpha$. Force is determined by:

$${\vec{\boldsymbol{F}}}_{flow}=\left[ \begin{aligned} L \\ D \end{aligned} \right]=\frac{1}{2}\rho S\left| v \right|^{2}\left[ \begin{matrix} C_{l}\left( \alpha_{0}+ \Delta\alpha\right) \\ C_{d}\left( \alpha_{0}+ \Delta\alpha\right) \end{matrix} \right]$$

$${\vec{\boldsymbol{F}}}_{global}={\vec{\boldsymbol{F}}}_{flow} R\left( \Delta\alpha\right)$$

The equation of motion is:

$${\vec{\boldsymbol{F}}}_{global}=\left[ \begin{aligned} -mg+ma_{z,bird} \\ ma_{x, bird} \end{aligned} \right]$$

***Sensitivity of gust rejection to potential error in the aerodynamic polar***

Our results are insensitive to systematic error in the CFD-derived aerodynamic polar. For this analysis we only examined the average relative contribution of inertial and aerodynamic rejection at the instant of peak inertial rejection. A 50%increase in drag decreased inertial rejection from 32% to 29% and increased aerodynamic rejection from 6% to 15%. The inertial rejection was still double aerodynamic rejection. Similarly, increasing the lift curve slope by even 20% over the linear portion (<10°) decreased inertial rejection by just 2% (to 30%) and increased aerodynamic rejection by 6% (to 12% of the total rejection). Increasing lift across the whole aerodynamic polar demonstrates that it is more sensitive to results at higher angle of attack, but even applying a moderate increase still did not affect our conclusions. We applied a 6.5% increase in lift coefficient—the amount required to achieve 100% weight support in the CFD simulations at 5 degrees angle of attack (the observed orientation and at the observed speed), but treated as systematic error across the whole aerodynamic polar— and this decreased inertial rejection from 32% to 27% and increased aerodynamic rejection from 6% to 21%. Even with this systematic increase in lift applied across the polar, inertial rejection was still 30% more effective than aerodynamic rejection at its peak.

***Centre of percussion calculation***

The centre of percussion $P$ derives from the wing mass distribution. It is given by

$$P=\frac{I_{h}}{m_{w}l_{G}},$$

where $m_{w}$ is the mass, $I_{h}$ the moment of inertia, and $l_{G}$ the centre of mass of the wing (the latter two being taken about the shoulder).

The wing CT-scan data comprise thousands of elemental voxels of identical volume $\delta V$, each with a unique position $\boldsymbol{p}\left( x,y,z \right)$ and density $\rho$. $x$ points along the shoulder hinge axis, $y$ along the wingspan, and $z$ in the dorsoventral direction. We obtain the mass of each voxel as $\delta m=\rho\delta V$, from which the inertial properties are obtained from the following sums:

$$m_{w}=\sum\delta m,$$

$$I_{h}=\sum r^{2}\delta m,$$

$$l_{G}=\frac{\sum r\delta m}{m_{w}}.$$

$r$ is the distance from the shoulder hinge to each voxel once projected onto the plane of rotation. By substitution, we find that the centre of percussion is

$P=\frac{\sum r^{2}\delta m}{\sum r\delta m}.$

***Centre of percussion derivation***

Gust force on the wing is transmitted to the torso as reaction at the shoulder hinge. If the gust force acts through the centre of percussion, though, initial reaction on the torso will be zero. We present here a simple, intuitive explanation of how this works.

Imagine a pendulum-like bar (thick grey line) whose pivot $O$ is free to translate sideways. If we push the bar transversely with force $F$ at the centre of mass $G$, it will accelerate linearly, without rotation, by amount $a_{G}$. The dashed green line shows the position of the bar an instant later (figure: left).

$$a_{G}$$

$$G$$

$$F$$

$$O$$

$$l_{G}$$

$$a_{O/G}$$

$$a_{G}$$

$$G$$

$$F$$

$$O$$

$$P$$

$$l_{G}$$

If we push the bar lower down, however, we can cause anticlockwise rotation about $G$. This accelerates $O$ leftwards relative to $G$ by amount $a_{O/G}$, in opposition to $a_{G}$ (which has the same sense and value as before because $F$ is identical). Now, there exists a special point for which these initial accelerations cancel, ‘the sweet spot’: the pivot remains still and the bar moves in pure rotation about $O$. The solid green line shows the bar in this condition at the same time instant as before (figure: right). Let us proceed to find ‘the sweet spot’:

The linear equation of motion for the bar is

$$F=ma_{G}.$$

The moment equation about $G$ is

$$\sum M_{G}=F\left( P-l_{G} \right)=I_{G}\ddot{\theta},$$

where $\theta$ denotes the rotation angle of the bar. By the notation above, we may now write the linear acceleration of the pivot $O$ as

$$a_{O}=a_{G}+a_{O/G}.$$

In other words, the motion of $O$ is that at $G$ plus that between $O$ and $G$.

We require $a_{O}=0$, or $a_{O/G}=-a_{G}=-F/m$. As mentioned, $a_{O/G}$ is the leftward (negative) acceleration of $O$ relative to $G$ by rotation, which is simply $-l_{G}\ddot{\theta}$ while angle $\theta$ is small. Thus $-l_{G}\ddot{\theta}=-a_{G}$, so (we cancel the minus signs)

$$\frac{Fl_{G}\left( P-l_{G} \right)}{I_{G}}=\frac{F}{m}.$$

By the parallel axis theorem ($I_{O}=I_{G}+ml_{G}^{2}$), after expanding brackets, we then find

$$mPl_{G}-ml_{G}^{2}=I_{O}-ml_{G}^{2},$$

which ultimately yields

$$P=\frac{I_{O}}{ml_{G}}.$$

Location $P$ is the ‘sweet spot’. It is known as the *centre of percussion*. A force at this point will not accelerate the pivot initially.

The idea also applies to a hinged wing. For a more detailed treatment of the problem, please refer to the subsequent derivation.

--

We now present a linearised model, comprising a point mass (torso) and two rigid beams (wings) moving in the plane, that demonstrates, for those interested in further detail, the same idea but for a bird-like system. The linearisation makes the dynamics more accessible, with the caveats that (i) the wing stay below 15-20 degrees, and (ii) the flow remain broadly attached to the lifting surface.

| **Term** | **Description** | **Positive sense** |
| --- | --- | --- |
| $m_{t}$ | Torso mass | --- |
| $m_{w}$ | Wing mass | --- |
| $I_{G},I_{h}$ | Moment of wing inertia, as given | --- |
|  |  |  |
| $l_{G}$ | Distance from hinge to centre of wing mass | --- |
| $l_{F},l_{F^{'}}$ | Distance from hinge to centre of pressure, as given | --- |
|  |  |  |
| $F,F^{'}$ | Aerodynamic force on each wing | Up |
| $R{,R}^{'}$ | Vertical reaction force between each wing and torso | Up |
| $T_{h},T_{h}^{'}$ | Hinge torque | Clockwise |
|  |  |  |
| $a_{t}$ | Linear vertical acceleration of centre of torso mass | Up |
| $a_{w}$ | Linear acceleration of centre of wing mass | Up |
| $z$ | Vertical torso position | Up |
| $\theta$ | Angular wing elevation from horizontal | Up |
| $P$ | Distance from hinge to centre of percussion | --- |

An exploded free body diagram of the model is shown below. Only one wing is drawn; the forces and geometry are the same on both sides. We omit horizontal forces because they are not relevant here.

$$\theta$$

$$T_{h}$$

$$T_{h}$$

$$T_{h}$$

$$m_{w}g$$

$$F$$

$$m_{t}g$$

$$R$$

$$R$$

$$R$$

$$z$$

$$l_{F}$$

$$l_{G}$$

**Figure**: Free body diagram of the model. Left: ‘point’ torso. Right: rigid wing.

The equation of motion for vertical force on the torso is

$$2R-m_{t}g=m_{t}a_{t}=m_{t}\ddot{z}.$$

We use $z$ for vertical torso position. For each wing, the linearised equation is

$$F-R-m_{w}g=m_{w}a_{w}=m_{w}\left( \ddot{z}+l_{G}\ddot{\theta} \right).$$

It is linear because $a_{w}$ is approximated as $\ddot{z}+l_{G}\ddot{\theta}$. The moment equation about the wing centre of mass $G$ is

$$F\left( l_{F}-l_{G} \right)-T_{h}+Rl_{G}=I_{G}\ddot{\theta},$$

We may substitute the force equation, along with $a_{m}=\ddot{z}+l_{G}\ddot{\theta}$ and the parallel axis theorem ($I_{h}=I_{G}+m_{w}l_{G}^{2}$) to obtain

$$Fl_{F}-T_{h}-m_{w}l_{G}g=m_{w}l_{G}\ddot{z}+I_{h}\ddot{\theta}.$$

Now, before the gust strikes, all time-dependencies disappear and each term assumes its equilibrium (0) value. We thereby obtain the equations of static equilibrium,

$$2R_{0}-m_{t}g=0,$$

$$F_{0}-R_{0}-m_{w}g=0,$$

and, for moments,

$$F_{0}l_{F0}-T_{h0}-m_{w}l_{G}g=0.$$

This is the steady level flight condition. If we subtract equilibrium forces, as is usual for linear systems, then

$$2\left( R-R_{0} \right)=m_{t}\ddot{z},$$

$$\left( F-F_{0} \right)-\left( R-R_{0} \right)=m_{w}\ddot{z}+m_{w}l_{G}\ddot{\theta},$$

and, finally,

$$\left( Fl_{F}-F_{0}l_{F0} \right)-\left( T_{h}-T_{h0} \right)=m_{w}l_{G}\ddot{z}+I_{h}\ddot{\theta}.$$

Notice that all bracketed terms now denote increments about equilibrium. We recast these with a prime (‘).

|  | $2R^{'}=m_{t}\ddot{z},$ | (1) |
| --- | --- | --- |
|  | $F^{'}-R^{'}=m_{w}\ddot{z}+m_{w}l_{G}\ddot{\theta}$ | (2) |
|  | $F^{'}l_{F^{'}}-T_{h}^{'}=m_{w}l_{G}\ddot{z}+I_{h}\ddot{\theta}$ | (3) |

The key point here is that, with the linear model, we can subtract static loads and deal only with increments. By doing this, we remove the influence of gravity from the model.

The fact that $Fl_{F}=F_{0}l_{F0}+F^{'}l_{F^{'}}$ is not immediately obvious. If $f$ denotes the distributed force that makes up $F$, then, by the centroid formula,

$$Fl_{F}=\int_{0}^{b} fydy.$$

$b$ is the wing semi-span. But $f=f_{0}+f'$ (total = static + gust) in our linear model, so we deduce

$$Fl_{F}=\int_{0}^{b} f_{0}ydy+\int_{0}^{b} f'ydy=F_{0}l_{F0}+F^{'}l_{F^{'}},$$

as required. Now, putting $\ddot{z}$ from (1) into (2) and rearranging, we find

$$\ddot{\theta}=\frac{F^{'}}{m_{w}l_{G}}-\frac{R^{'}}{m_{w}l_{G}}-\frac{2R^{'}}{m_{t}l_{G}}.$$

When inserted into (3), this gives

$$F^{'}l_{F^{'}}-T_{h}^{'}=m_{w}l_{G}\left( \frac{2R'}{m_{t}} \right)+I_{h}\left( \frac{F^{'}}{m_{w}l_{G}}-\frac{R^{'}}{m_{w}l_{G}}-\frac{2R'}{m_{t}l_{G}} \right)$$

(We have also substituted $\ddot{z}$ using (1) here.) Collecting force terms,

$$F^{'}l_{F^{'}}-T_{h}^{'}=R'\left( \frac{2m_{w}l_{G}}{m_{t}}-\frac{I_{h}}{m_{w}l_{G}}-\frac{2I_{h}}{m_{t}l_{G}} \right)+F'\left( \frac{I_{h}}{m_{G}l_{G}} \right).$$

The red portion contains only inertial properties and is constant for any given flyer. We absorb it as new constant $K$. After rearrangement,

$$R^{'}=\frac{F'}{K}\left( F^{'}l_{F^{'}}-\frac{I_{h}}{m_{w}l_{G}} \right)-\frac{T_{h}^{'}}{K}.$$

Complete torso stabilisation (zero acceleration) requires that the reacted force increment $R’$ be eliminated. Setting $R’=0$ and solving for $l_{F^{'}}$ yields

$$l_{F^{'}}=\frac{I_{h}}{m_{w}l_{G}}+\frac{T_{h}^{'}}{F'}.$$

In the classic case of the ‘free’ hinge under zero torque, the necessary condition is simply

$$l_{F^{'}}=\frac{I_{h}}{m_{w}l_{G}}=P.$$

In other words, $F’$ must act at a specific point located $l_{F^{'}}=P$ from the wing root. This point is known as the *centre of percussion*. It is an inertial property and, as such, constant for a given wing mass distribution.

For the owl, $l_{F^{'}}=P$ works only at first. Once $T_{h}^{'}$ and $F$’ develop, stabilisation requires that

$$l_{F^{'}}=P+\frac{T_{h}^{'}}{F'}$$

i.e. that the gust force act beyond the centre of percussion by an amount equal to the force-normalised hinge torque.

Note: in mechanical systems, $T_{h}’$ is usually produced by springs and dampers (passive) or prescribed by actuators (active). Here, mechanical elements are replaced by muscles, tendons, etc.
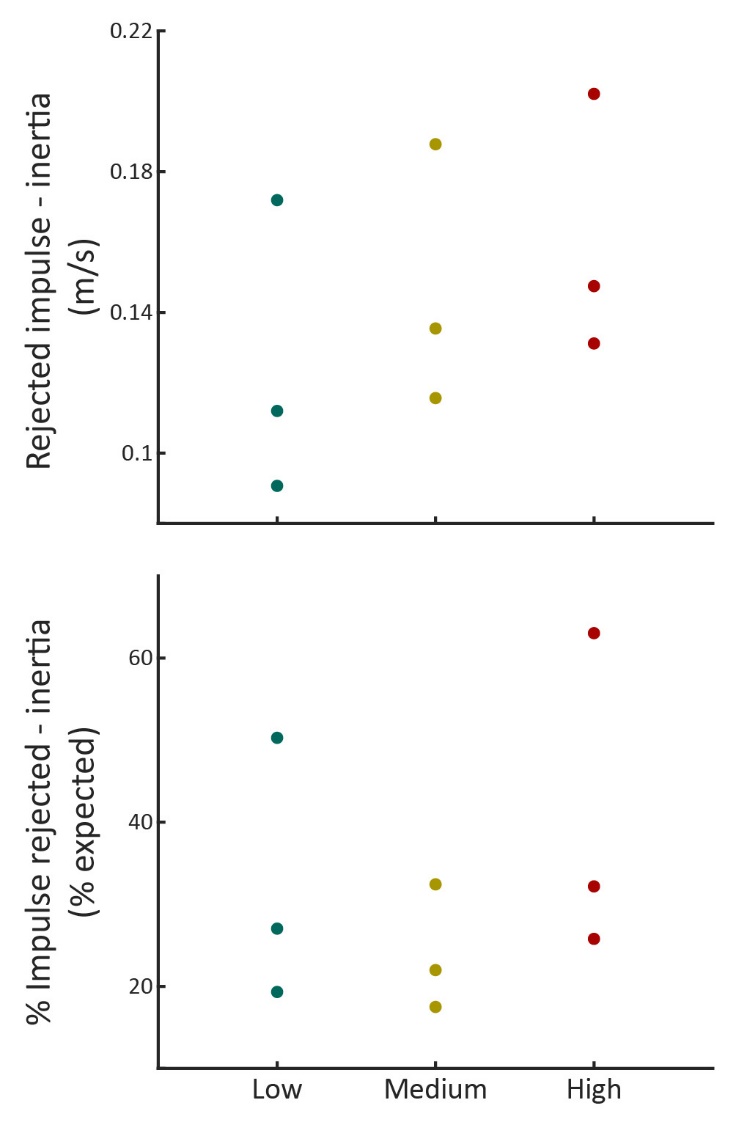


**Figure S1.**

**Inertial gust rejection: absolute and relative rejected impulse versus gust intensity.** Peak rejected impulse tended to increase with gust intensity, but did not discernably differ across gust intensities when normalised by the total expected gust impulse.


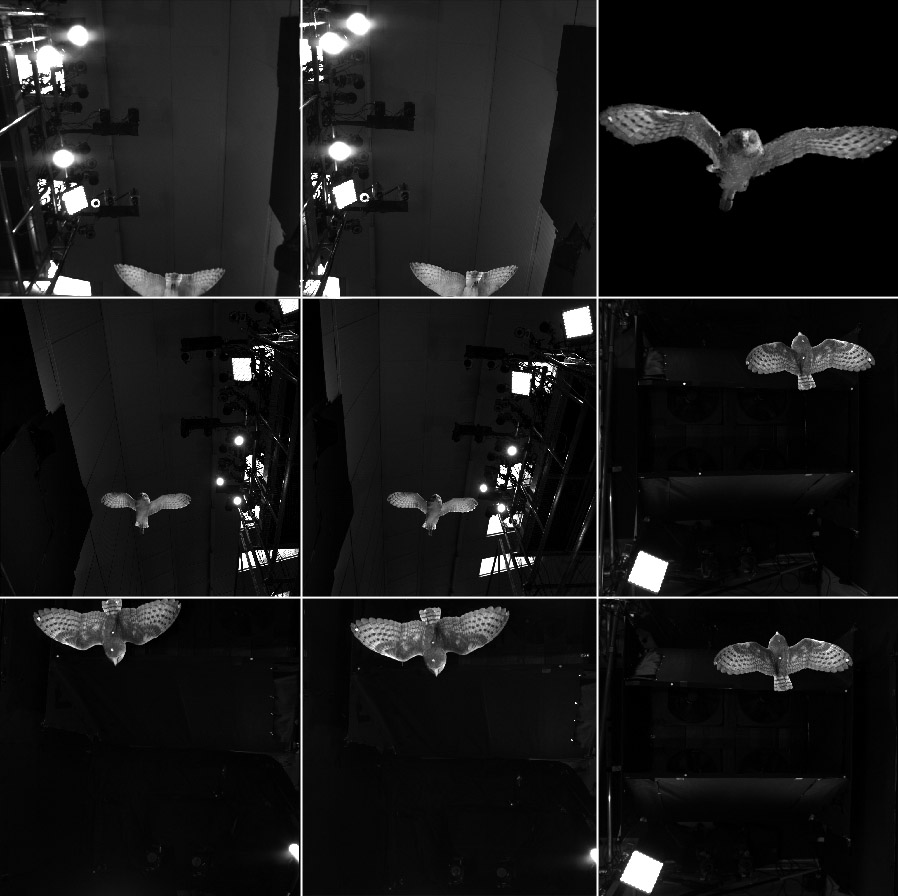


Figure S2.

**Example images from high speed video cameras.** Five pairs of high-speed cameras (two on the scaffold tower, three on the ground) provided full coverage of the measurement region. Typical views of the bird inside the gust are shown. The upper right-hand panel displays the point-cloud reconstruction at this moment. Not shown are views from one pair further downstream, which at this moment do not capture the owl.


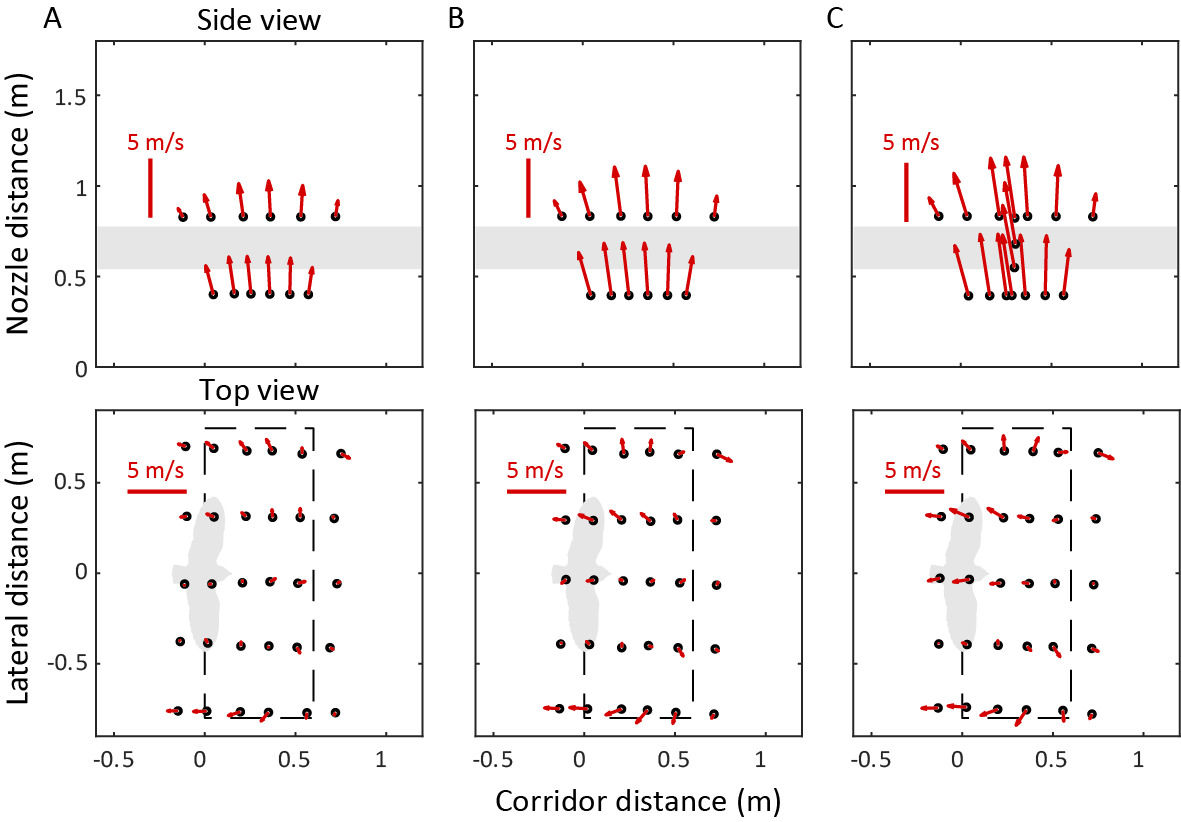


Figure S3.

**Measured mean gust velocity fields.** Side (span-averaged, two-planes) and top-down (upper-plane) views of the mean velocity vector fields for low (A), medium (B) and high (C) gusts show consistency and smoothness. The red scale bars define vector length. Also note that here the corridor origin is fixed to the upstream edge of the nozzle outlet. All flights paths lay within the grey band.


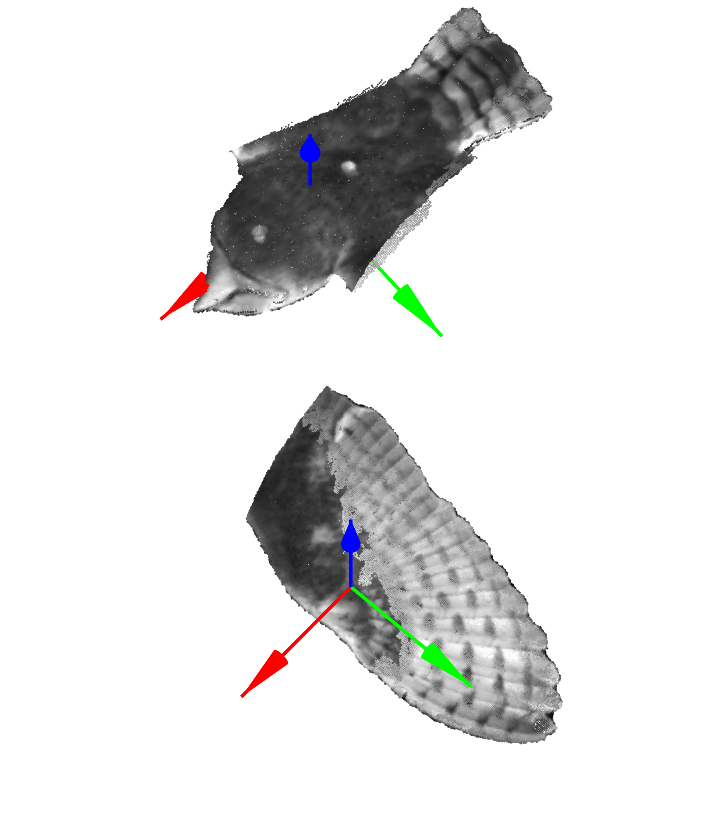


Figure S4.

Wing and body coordinate system. Rotations of the wing are determined as deviations between the coordinate systems of the body and the wing. All rotations are zero if the two coordinate systems run parallel. Rotation matrices were converted to Euler angles for wing movement description and computed in the order: sweep (Z), elevation (X’), and pitch (Y’’). This rotation order differs from the more common Z-Y’-X’’ to preserve long-axis rotation as the final computed rotation.
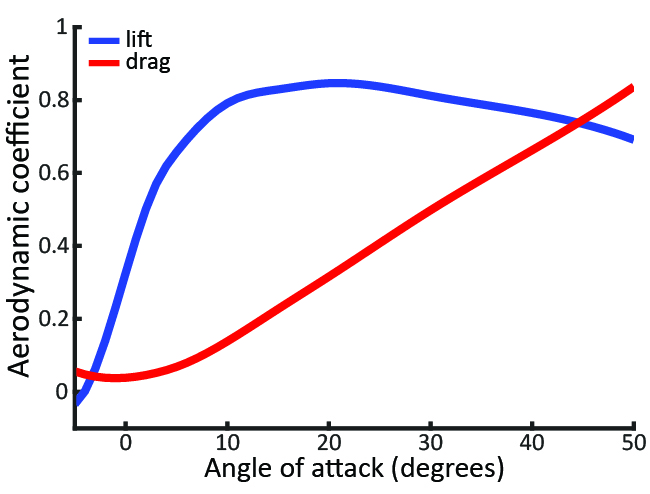


Figure S5.

**Lift and drag coefficients for a rigid bird derived from CFD modelling.** The lift coefficient (blue) decreases slightly at high angles of attack, demonstrating relatively soft stall characteristics. Drag coefficient (red) rises modestly as angle of attack increases.


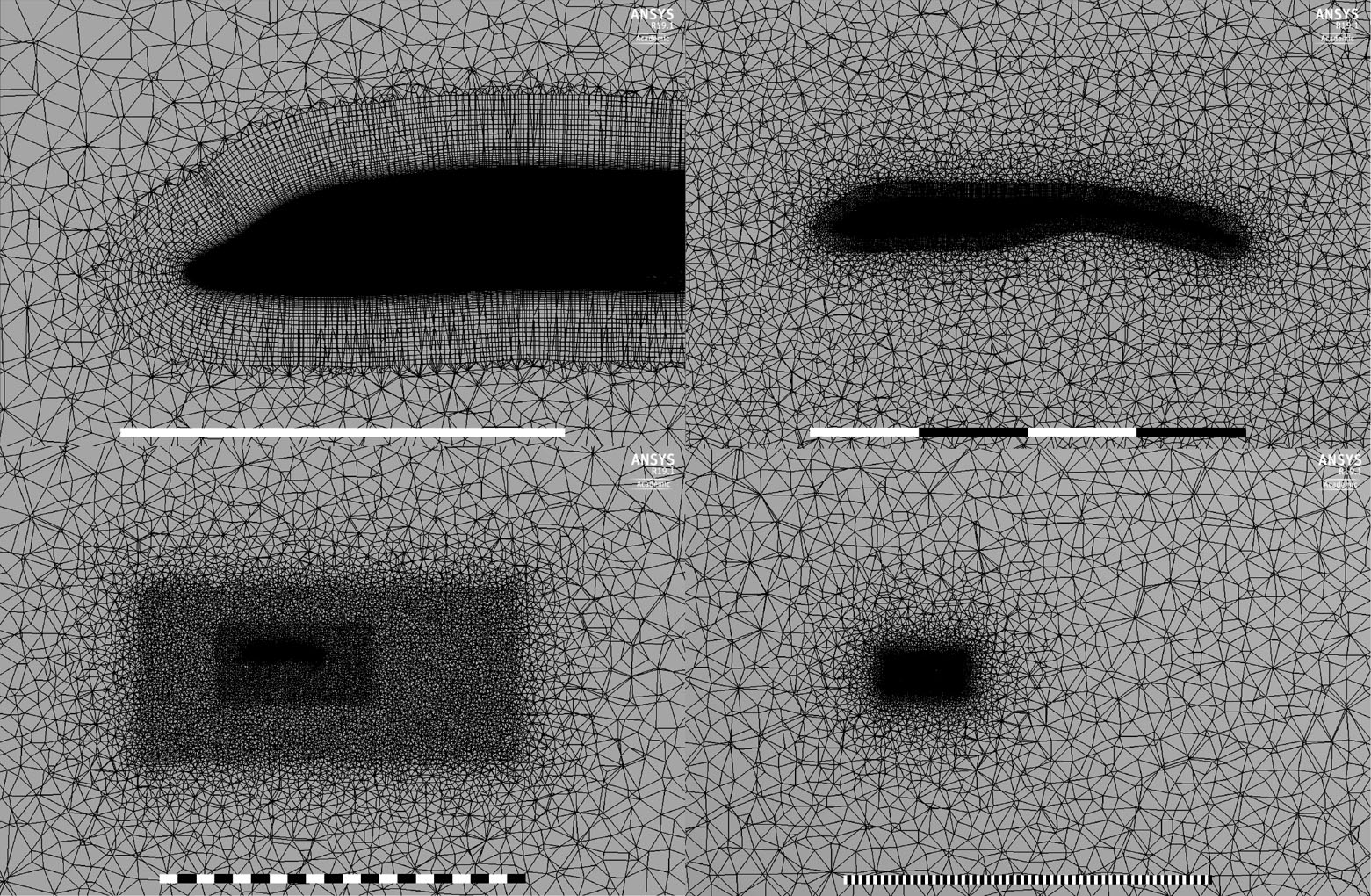


Figure S6.

**CFD mesh around the wing.** A slice through the wing at four different scales, showing the inflation layers at the wing surface, the variable bounding regions and the far-field (for quick but accurate computation). White/black bars are 5 cm long.

Movie S1 (separate file).

Frontal high-speed footage (recorded at 1000 fps, playback at 30 fps) of the owl shows the torso stabilisation effect during a high-intensity gust. Notice the delay between angular wing elevation and downward pitching.

Movie S2 (separate file).

The kinematic response to the gust scaled with gust intensity. Frontal views of the 3D-reconstructions of the bird responding to the gust demonstrate the full kinematic response. All 3D-reconstructions are referenced to approximately the midpoint of the shoulder, but allowed to freely pivot about that point. The point clouds are tinted based on gust intensity: blue-control, green-low, yellow-medium, red-high.

Movie S3 (separate file).

Wing morphing is negligible until after peak inertial rejection. The perimeter of the left wing at two instances for each flight: pre-gust, and the moment of peak inertial rejection. The wings have been aligned using an ICP algorithm and colour-coded by gust intensity: green-low, yellow-medium, red-high.

Movie S4 (separate file).

Approach postures were consistently similar across gusted and control trials, suggesting no difference in behaviour before or between perturbations. The solid bird geometry (red) used in CFD was representative in this study.

Movie S5 (separate file).

In the control flights, the bird adopted and maintained a neutral glide posture throughout the measurement region. Body and wing orientation were similar across the thirteen trials, with some variation in magnitude and symmetry of wing dihedral. The solid bird geometry (red) used in CFD was representative in this study.

Dataset S1 (separate file).

Excel data file containing raw data for: lift/drag polar from CFD, data for initial conditions of rigid gliding flight simulations, wing mass and lift distributions, and kinematic data for all flight trials.
